# Roots and Wings: The Role of Job Embeddedness in Mitigating Nurses' Emigration Intention Through a Serial Mediation Lens

**DOI:** 10.1155/jonm/9917479

**Published:** 2025-09-29

**Authors:** Akinwuyi Stephen Akinwande, Cem Tanova, Steven Bayighomog, Deborah Onaopemipo Ajayi

**Affiliations:** ^1^Faculty of Economics and Administrative Sciences, Cyprus International University, Nicosia, North Cyprus, Turkey; ^2^Department of People & Organizations, Bournemouth University, Poole, UK

**Keywords:** employee engagement, employee voice, intention to emigrate, job embeddedness, nurses

## Abstract

**Aim:**

This study investigates the influence of job embeddedness on nurses' emigration intentions with a focus on the mediating roles of employee voice and engagement.

**Background:**

The migration of healthcare professionals from developing to developed countries exacerbates the brain drain crisis, posing challenges to healthcare systems in low-income countries. Nurses may be less likely to have emigration intentions if they have higher levels of job embeddedness in their workplaces. However, limited research has explored this relationship in low-income contexts.

**Methods:**

Drawing on Lewin's field theory and the theory of the cumulative causation of migration, a cross-sectional study was conducted using survey data from 336 nurses in Nigeria. SmartPLS 4.0 was used to analyze the direct and indirect effects.

**Results:**

Findings revealed a negative relationship between job embeddedness and emigration intentions mediated by employee voice and engagement.

**Conclusion:**

The study provides important contributions to management of healthcare organizations and migration research and offers practical implications for policymakers, service sector managers, and future research.

## 1. Introduction

As global emigration patterns grow more complex, the healthcare industry faces a critical shortage of skilled professionals, particularly nurses, as wealthier countries increasingly rely on foreign-trained workers to meet demands [1]. For instance, of the 29.8 million total global stock of nurses, the 2025 world's nursing report released by the WHO [2] revealed that 11% of practicing nurses were foreign-trained, and 16% of these nurses were in high-income countries. This migration flow exacerbates workforce shortages in low- and middle-income countries, undermining their health systems and deepening healthcare inequalities [3–5]. In the low-income countries, particularly across Africa, the emigration of nurses has attracted serious concerns about the sustainability of domestic healthcare systems [6]. Nigeria, for instance, faces a significant brain drain, with significant number of nurses leaving each year in search of better job opportunities in high-income countries [7]. This exodus is driven by factors such as economic disparity, poor working conditions, and limited career growth prospects [5, 8, 9]. As a result, these countries face a growing shortage of skilled healthcare professionals, severely impairing their ability to deliver quality care [10].

The theory of cumulative causation of migration framework supports this outcome through the hypothesis of the “migrant syndrome,” which creates a vicious migration cycle leading to more underdevelopment, fueling further migration [11]. Given the unsustainability the migration flow perpetuates on domestic countries' healthcare systems' human capital supply, the theory recognizes structural endogenous and exogenous mechanisms which can halt or inhibit the migration dynamics. As it stresses macro and meso factors, it fails to account for micro factors at the individual level, such as personal agency or psychological levers, which can significantly deter individuals' migration behaviors. In this sense, place attachment has recently emerged as a pull/push idiosyncratic factor that could determine staying or emigrating intentions [12, 13]. However, this association is somewhat unclear as it is limited to a framework [12] or without empirical support with a sample of high school students [13]. A robust empirical investigation focusing on the working population, which accounts for about 59% of total migrants [14], is therefore warranted.

Against this backdrop, the present study builds on the concept of job embeddedness to propose a research model that predicts a decrease in healthcare workers' emigration intentions. The empirical literature on job embeddedness supports that it is strongly associated with employee retention [15] because it alleviates employee turnover intention [16–18], intention to quit [19], intentions to leave, and actual voluntary leaving [20]. Although there is little insight into whether job embeddedness potentially explains healthcare professionals', such as nurses', rationale to emigrate to other countries [21, 22], embedded employees are relevant assets for organizations to be retained [23]. Furthermore, the mechanisms underlying the effects of job embeddedness on emigration intentions among nurses remain unexplored. This study explores psychological empowerment and positive organizational behaviors resulting from increased job embeddedness, namely, employee voice and engagement, as significant factors in the underlying pathways of job embeddedness-emigration intention association. Understanding job embeddedness and its relationship with intention to emigrate through employee voice and engagement can offer healthcare service providers, administrators, and policymakers relevant insights into healthcare professionals' employment stability and retention outcomes.

From a scholarship perspective, this study delivers empirical insights into the taxonomy of mitigating drivers (“pull” factors) of emigration intentions that have been paradoxically overlooked in lieu of “push” factors (e.g., [24]). Hence, it contributes to bridging the job embeddedness and emigration literature. From a policy perspective, exploring and leveraging the relevance of “pull” factors is crucial for two correlated reasons. First, typical “push” factors to emigration intentions, such as corruption [25], low remuneration, and inadequate infrastructure [10, 24], are generally more prominent in low-income countries, which can hardly compete with the appealing opportunities offered by high-income countries. Second, local policymaking stakeholders can tap into “pull” factors as preliminary levers that could precede or complement “push” factors, which can be challenging, time-consuming, and resource-hungry to curb drastically. Moreover, this study can support ministries of health, Nursing and Allied Health Associations, International Organizations, and Nongovernmental Organizations, in articulating strategies for planning, implementing, and improving regulatory frameworks, such as the Global Strategy on Human Resources for Health: Workforce 2030 [26] and Nursing and Midwifery Council (NMC) Regulations, aligning them with Sustainable Development Goals (SDGs) 3 and 8—which, respectively, address the support for healthy lives and well-being and decent work.

### 1.1. Theoretical Framework

The theory of cumulative causation of migration [27] provides strong explanations for how these extensive migration processes create self-reinforcing patterns through expanding social networks.  The presence of strong migrant connections within communities leads to increased emigration because the path becomes both physically and socially accessible [28]. The act of migration then evolves from a risky decision into a strategic choice with no signs of stopping [28, 29]. While this theory is structural in nature, it appears to overlook personal agency as a disruptive force that can halt or slow the migration cycle. These forces include personal values such as family commitment [30], psychological factors such as self-efficacy [31], and improved conditions such as better wages [32]. This study contends that job embeddedness, a psychosocial variable, may halt the emigration intention. Employees who feel rooted through being valued, connected, and heard will experience a transformation in their social capital. The networks that previously encouraged their migration may now function as forces that discourage them from emigrating. The migratory stream decreases when individuals develop psychological resistance and personal agency (such as voice and engagement) against the intention to emigrate.

The second theory, Lewin's field theory, works on the principle of behavior (*B*) being a function of person (*P*) and their psychological environment (*E*) [33, 34]. The standard mathematical expression used for this theory is *B*=*f*(*P*, *E*) [33, 34]. In this framework, voice and engagement are proximal elements of the environment, which are psychologically desirable features of the employees' experience and can produce the most potent effect on behavior. More distal features, such as job embeddedness, frequently influence behavior directly or indirectly, through its influence on how employees perceive proximal elements. The accurate representation of individual behaviors develops from the cognitive map, which combines proximal and distal forces that affect people within the field [35, 36].

### 1.2. Relationship Between Job Embeddedness and Intention to Emigrate

Individuals who migrated at some point felt a weaker sense of attachment to the organization [37], which later transformed to actual emigration. This may illustrate a low level of job embeddedness, while those who have remained may be highly embedded. Meanwhile, nurses who emigrate are often in constant touch with their colleagues left behind in their home country. With insight from the theory of cumulative causation of migration, how migration creates a self-sustaining process through the connections between emigrants and people who remain at home is explained [38]. The growing number of nurses who leave their jobs creates stronger connections which in turn drive more nurses to emigrate. The macro-level patterns described by this theory, such as wage gaps and chain migration, receive effective explanation, but the theory fails to consider individual factors that could interrupt this pattern [39]. Job embeddedness becomes essential because nurses who maintain strong workplace and community connections may overcome greater personal obstacles when thinking about emigrating. Besides, the structural pressures (such as exhaustion and job satisfaction [38, 40]) that drive weakly embedded nurses to seek better opportunities abroad may not affect nurses who have strong job embeddedness because they tend to stay. Job embeddedness, as suggested by Lewin's field theory, can function as a micro-level opposing force to the macro-level migration dynamics described by theory of cumulative causation of migration. Accordingly, by enhancing the perceived costs linked with emigration, a high level of job embeddedness can hamper the brain drain cycle by decreasing the push-to-leave factors' effect [41]. That is, highly embedded nurses can become reluctant to dissolve bonds with jobs, coworkers, and community, renounce the emotional investment made on patients, or draw away from the cultivated professional associations. Hence, it is proposed that:

• H1: Job embeddedness has a negative relationship with intention to emigrate.

### 1.3. Relationship Between Job Embeddedness, Employee Voice, and Intention to Emigrate

Embedded nurses are apt to demonstrate voice behavior, such that they can be willing to share ideas, suggestions, or concerns regarding unit decision making, safety, and the improvement of work environment or patient care [42, 43]. The willingness of nurses to exhibit voice behavior arises from their workplace resilience which evolves through supportive social networks [44]. The networks, such as trust and mutual support, protect the nurses from negative outcomes of speaking up, thus making them nonsusceptible toward the outcomes of their voice behavior. Besides, high job embeddedness most often offers employees career success and job security [45] which acts as impulse to speak up. Moreover, when voice cost is low and employees perceive that their voice can impact feasible changes, their vested interest for organizational change increases, making them to become motivated to make suggestions for improvement [46]. This implies that when the employee voice is perceived to be appreciated by the organization and unconditionally expressed by the employees, the more the feeling of meaning and the desire to make a difference to their job and organization. This could deepen their attachment and inhibit withdrawal cognitions such as quitting and emigration intention. Furthermore, aligning with the aforementioned arguments, it is suggested that job embeddedness determines employee voice congruent to solving problems, thereby reducing intention to emigrate. Drawing on Lewin's field theory, the mediation role of employee voice is elucidated. Based on this theory, job embeddedness generates binding forces which can keep nurses connected to their work roles [20, 47]. The effectiveness of voice in communication strengthens the retaining forces of job embeddedness [48] because it enables employees to feel engaged and in control while maintaining hope, which may increase retention regardless of push factors [49]. The suppression of voice may weaken the binding power of job embeddedness, leading to increased feelings of servitude and insignificance [48]. This transforms the inherent normality of job embeddedness into dissatisfaction, directly increasing emigration intention by redirecting the equilibrium toward exit. Employee voice, thus, may determine whether job embeddedness functions as an anchor or creates frustration, which mediates its eventual impact on emigration intention. Employee voice has often been identified as a potential mediator. For instance, past studies have established organizational behavior and workplace dynamics variables (i.e., servant leadership and Industrial relations climate) indirectly linked to turnover and career mobility variables (i.e., turnover intention, intention to resign, and intention to quit) through employee voice [50, 51]. Similarly, this study expects employee voice to mediate the job embeddedness and intention to emigrate relationship. Hence, it is proposed that:

• H2: Employee voice mediates the relationship between job embeddedness and intention to emigrate.

### 1.4. Relationship Between Job Embeddedness, Employee Engagement, and Intention to Emigrate

Job embeddedness underscores the stability [52] and attachment [53] that encourages employees to direct their efforts to their work. This stability and attachment to the organization can occasion employee engagement [54]. When employees perceive harmony between their career goals, organization's goals, getting the support of their coworkers, and acknowledgment of the cost implication of leaving, they tend to get more engaged. In other words, as job embeddedness increases, engagement among the employees may increase. Although the literature on the relationship between employee engagement and intention to emigrate is scarce, few studies (e.g., [55, 56]) indicated that engaged employees have higher intention to remain in their employing organization leading to lower turnover. This implies that employees with high engagement level easily express themselves using their physical, cognitive, and emotional states while performing their workplace tasks [57]. Consequently, they tend to show less intention to emigrate. Since a negative relationship between employee engagement and turnover intention has been reported by extant studies, we can assume that employee engagement may be negatively related with intention to emigrate. According to Lewin's field theory, which captures the emigration intention as the product of the relationship between opposing forces, intention is attributable to the interaction between the individual and the environment [58]. This is directly influenced by opposing forces which either exert on individual a push-to-leave or pull-to-stay toward an intended decision. Exhaustion and job dissatisfaction from low voice and engagement [40, 59] among others are the push-to-leave (driving) forces, while the pull-to-stay (restraining) forces include job embeddedness [60, 61], engagement, and voice behavior [62]. These forces often affect the retention of nurses, support fulfillment, and a supportive work environment [63–66]. Synthesizing the above arguments, employee engagement may mediate the relationship between job embeddedness and intention to migrate. Gathered evidence reveals that job embeddedness can predict engagement, indicating that employees with high job embeddedness will be more engaged [67]. As a result, intention to emigrate may be reduced due to the high employee engagement. Accordingly, it is proposed that:

• H3: Employee engagement mediates the relationship between job embeddedness and intention to emigrate.

### 1.5. Relationship Between Job Embeddedness, Employee Voice, Employee Engagement, and Intention to Migrate

In situations when nurses' emigration intention increases on the account of push-to-leave forces, employee voice and engagement may address the concerns of the employees, through a sense of inclusion, value, and influence within the organization [68], which may support the reasons to give up their emigration intention [69]. The integration of Lewin's field theory and the theory of cumulative causation of migration reveals how strong job embeddedness decreases nurse emigration intention through employee voice and engagement. Nurses who feel deeply embedded in their work environment because of strong ties, organizational fit, and meaningful work experience feel psychologically safe to express their opinions. In healthcare practice, a sense of value is apparent via the strength of voice recognition regarding patient care, workplace conditions, and healthcare practices [70, 71]. Besides, when organizations embrace the culture of open communication, employees perceive the sense of inclusion and value, thereby giving them influence within the organization [68]. When this perception is consistent, the typical outcome is an increase in engagement of employees [68, 72], which may counterbalance the intention to emigrate. The collective sharing of empowerment stories and visible workplace satisfaction transforms social norms in a way that staying at work becomes more desirable than leaving. The continuous positive feedback loop between staying and leaving work increases the social cost of leaving while creating multiple psychological advantages of staying, transforming personal retention into a widely held norm. Hence, we propose that:

• H4: Employee voice and employee engagement serially mediate the relationship between job embeddedness and intention to emigrate.

**Figure 1:**
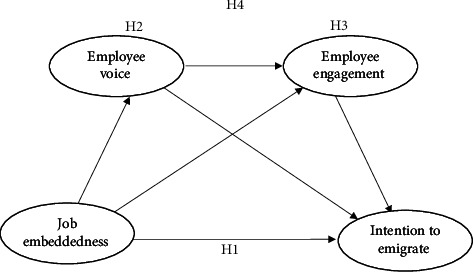
Research model.

Based on the developed hypotheses, the developed research model is presented in Figure 1.

## 2. Methods

### 2.1. Sample and Data Collection

The population of interest for this study consisted of practicing and licensed nurses working in government and private hospitals in Nigeria. The hard copies of the questionnaire were distributed to the nurses through the Chief Nursing Officers and nurse-organized groups such as the National Association of Nigeria Nurses and Midwives (NANNM) state chapters. The surveys were self-administered, and the respondents were informed of the academic purpose of the study, including a guarantee of anonymity and confidentiality of the use of the information provided. No personal information that could identify the participants was required. The institutional review board of the first author's institution granted ethical approval for the research. With the unknown population of nurses and their list unavailable [73], the sample size was statistically determined using the G∗power program [74]. A minimum sample size of 147 was determined at 15% effect size, 5% alpha, and 90% power with ten predictors. We used a convenience sampling procedure, prioritizing the respondents' comfort and embracing the abovementioned ethical considerations [75]. Factoring in the response rate and some other uncontrollable factors in this study, 369 responses were collected. About 33 copies were unusable, while 336 were usable and subjected to statistical observation. Among the 336 nurse respondents, 51.8% were female and 48.2% were male. About two-thirds of the respondents (67%) are within the age groups 30–39 years and 40–49 years. Respondents who were married, divorced, or widowed are 55.1% while those who were never married are 44.9%. The job tenure of the respondents varied; 33% had a tenure below 9 years and below while 77% had a tenure of 10 years and above. About four-fifths of the respondents (80.1%) have their highest education level as a diploma in Nursing and a Bachelor of Science. About 64.9% and 35.1% of the respondents were employees, respectively, serving in public and private hospitals. More than four-fifths of the respondents (82.1%) have over 11 years of service in their organization.

### 2.2. Measurement

All the items of the constructs were adopted from previous studies, and the instrument was prepared in English (Table A1). Three items of job embeddedness were adopted from Karatepe and Avci [76]. The employee engagement items were adopted from Schaufeli et al. [77]. The items were divided into three subscales: vigor, dedication, and absorption with each having three items. The mean of the responses to the nine items represented overall employee engagement. The six established items of employee silence were adopted from LePine and Van Dyne [78]. Finally, the three items of intention to emigrate were modified from Hoong and Soon [79]. All of the items were anchored on a 5-point Likert scale ranging from 1—strongly agree to 5—strongly disagree. The demographic profile of the respondents featured in this study includes gender, age, organizational tenure, level of education, marital status, and job title. They were also considered covariates of this study.

### 2.3. Analytic Strategy

This study used SPSS 22 to conduct descriptive analysis of the demographic profile and bivariate analysis. SmartPLS 4 [80] was also utilized to perform a two-step approach for partial least square structural equation modeling (PLS SEM). First, series of confirmatory factor analyses (CFAs) were run to assess the factorial validity of the measurement model. Then, a structural model was derived to test the proposed research model presented in Figure 1. The hypotheses for both direct and indirect relationships were tested by assessing the statistical significance at 95% confidence level, 10,000 bootstrap resamples-generated confidence intervals. The direct and indirect paths were statistically significant if their respective confidence interval did not straddle zero.

## 3. Results

### 3.1. Convergent Validity

This study inspected the data for convergent validity using PLS algorithm. Based on the recommendation of Hair et al. [81], this study assessed the validity through factor loadings, average variance extracted (AVE), and composite reliability (CR). As observed in Table 1, the items loaded above the value of 0.5, the threshold suggested by Hair et al. [81], were retained. The items that loaded below 0.5 were deleted, for instance, one item and five items of employee voice and employee engagement, respectively, fell below threshold. Besides, the CR was accessed. Using the estimate, the extent to which the items of each construct consistently reflected the investigated construct was determined with a threshold of 0.7 [81]. As shown in Table 1, all the CR values exceeded 0.7 (0.837–0.900) as recommended by Hair et al. [82]. Lastly, the AVE for all the constructs was above 0.5 (0.632–0.665) as recommended by Hair et al. [81].

### 3.2. Discriminant Validity

In this study, the discriminant validity was examined by comparing the correlation of each pair of latent constructs against the square root of the AVEs [83]. As shown in Table 2, the square root of the AVEs (the bold and diagonal values) was higher than each bivariate correlation, indicating discriminant validity.

Furthermore, the heterotrait–monotrait (HTMT) ratios were generated to support the discriminant validity. As suggested by Henseler et al. [84], the HTMT value below 0.9 suggests acceptable discriminant validity. As observed between constructs, the values are all below 0.9 which suggests that the constructs are satisfactorily distinct (see Table 3).

### 3.3. Common Method Variance (CMV)

To minimize CMV threat, the message and assurance of confidentiality and anonymity were conveyed. In addition, the purpose of the study was stated. To further detect the issues of CMV, we performed Harman's single-factor test using CFA. The results showed that a single factor model explained only 38% of the total variance, which is below the 50% threshold suggested by Shiau and Luo [85]. This suggests that common method bias does not present a serious threat to the study's findings. Additionally, a collinearity test was done to identify the presence of any variance inflation factor (VIF) above 3.3. After careful observation, all the VIFs in the inner model were below the value of 3.3 as suggested by Kock [86], an indication of little or no concern on CMV.

### 3.4. Predictive Power, Predictive Relevance, and Path Coefficients

The predictive power of the model of this study was measured via the *R*^2^, coefficient of determination.  Table 4 shows that job embeddedness explained 29.3%, 29.0%, and 49.0% variance for employee voice, employee engagement, and intention to emigrate, respectively. Using the Hair et al. [81] threshold, the *R*^2^ of the constructs reflects low predictive power. *Q*^2^ was used to estimate the out-of-sample prediction of the model. From Table 4, all the *Q*^2^ values were above 0 (0.129–0.284). According to Sarstedt et al. [87], a *Q*^2^ value above 0 reveals that the PLS model has a predictive relevance.

### 3.5. Bivariate Analyses

To examine the correlation between the independent variables, control variables, and dependent variable, bivariate statistical tests were carried out using Pearson correlation coefficients (Table 5). Among the seven demographic covariates, only gender (*r* = 0.110, *p* < 0.05), job tenure (*r* = −0.112, *p* < 0.05), and education (*r* = −0.164, *p* < 0.01) were significantly correlated with intention to emigrate. Even though the effect size of these correlations was small, they are considered as covariates in the subsequent analysis. Job embeddedness (*r* = −0.476, *p* < 0.01) was negatively and significantly correlated with intention to emigrate. Employee voice (*r* = −0.680, *p* < 0.01) and employee engagement (*r* = −0.449, *p* < 0.01) were also negatively and significantly correlated with intention to emigrate. In all, the main variables all have moderate effect size.

### 3.6. Test of Hypotheses

In this structural model (Table 4), the path coefficients were presented. There were five direct effects and three indirect effects which informed the eight hypotheses of this study. The relationship between the three control variables and intention to emigrate was tested. Findings showed that gender (*β* = 0.077, 95% CI = −0.057, 0.210), job tenure (*β* = −0.051, 95% CI = −0.116, 0.014), and education (*β* = −0.005, 95% CI = −0.074, 0.083) had no significant relationship with intention to emigrate.

#### 3.6.1. Direct Paths

The relationship between job embeddedness and intention to emigrate was negative and statistically significant (*β* = −0.134, 95% CI = −0.233, −0.049). The relationship between job embeddedness and the mediators was examined. Job embeddedness has statistical significance on employee voice (*β* = 0.541, 95% CI = 0.459, 0.606). Also, job embeddedness has statistical significance on employee engagement (*β* = 0.118, 95% CI = 0.027, 0.202). Lastly, the relationship between the two mediators and intention to emigrate was examined. The relationship between employee voice and intention to emigrate was negative and statistically significant (*β* = −0.545, 95% CI = −0.635, −0.431), and the relationship between employee engagement and intention to emigrate was negative and statistically significant (*β* = −0.111, 95% CI = −0.188, −0.038) (Table 4).

#### 3.6.2. Indirect Paths

The indirect relationship between job embeddedness and intention to emigrate through (a) employee voice was negative and statistically significant (*β* = −0.295, 95% CI = −0.364, −0.230); (b) employee engagement was negative but not statistically significant (*β* = −0.013, 95% CI = −0.029, −0). Job embeddedness and intention to emigrate have statistically significant serial indirect relationship through employee voice and engagement (*β* = −0.028, 95% CI = −0.050, −0.009) (see Table 4).

The total indirect effect of job embeddedness on intention to emigrate through the two proposed mediators was statistically significant (*β* = −0.336, 95% CI = −0.404, −0.272). The total effect of job embeddedness on intention to emigrate was negative and statistically significant (*β* = −0.469, 95% CI = −0.546, −0.398). Since employee voice and engagement in the serial mediation accounted for 70.67% of the total effect (see Table 5), these results reveal that job embeddedness alone does not predict nurses' intention to emigrate. Specifically, employee voice and engagement partially account for the variance between these two constructs.

## 4. Discussion

A typical consensus among past studies is that the strong connection of employees to their job and community usually engenders attachment and retention [88] which decreases turnover intention and intention to stay in their current job and organization [89, 90]. However, the need to investigate frameworks that will aid retaining skilled healthcare workers in the low-income countries' healthcare industry caused by brain drain has been the main objective of this study. This study is driven to analyze the direct and indirect relationship between job embeddedness and intention to emigrate in the Nigerian healthcare industry. This relationship has not received adequate attention by existing empirical studies especially in the low-income countries. Hence, this study was carried out in Nigeria. Nigeria is the most populous and poorest African nation in the world [91–94], which qualifies it as a low-income country. Additionally, due to the recent wave of international nurse emigration, nurses who emigrated from this country are reported to be the largest nationality in the UK NHS based on headcount [95].

In summary, job embeddedness, which reflects the extent to which employees feel connected to their jobs and communities, played a key role in predicting emigration intention, even after controlling for demographic variables (H1). This indicates that employees' gender, education level, and job tenure did not significantly influence the observed strong negative relationship between job embeddedness and emigration intention. The results of this study revealed that job embeddedness has a negative relationship with intention to emigrate. This finding suggests that nurses with higher levels of job embeddedness are more invested in their current roles and communities, leading to a lower intention to emigrate. This negative relationship further implies that embedded nurses prioritize their individual and social links and the stability of their current environment over the potential benefits of emigrating.

To provide for an increased understanding of factors influencing emigration intention in the healthcare industry, this study investigated the mediation role of employee voice in the job embeddedness and intention to emigrate relationship (H2). The result shows that employee voice significantly played a mediating role. Some studies have pointed out that employees with high job embeddedness are likely to have and demonstrate more constructive behaviors, such as voice, that are needed to improve and prosper the organization [43, 96]. Nurses will likely show little or no intention to emigrate when the cost of voice behavior is low. The results also reveal that job embeddedness has a positive and significant relationship with employee voice. This implies that nurses with high job embeddedness feel at ease to display voice behaviors, offering their input and expressing discontent with no adverse repercussions. This is in tandem with some past studies [48, 96]; they noted that having employees with high job embeddedness can benefit the organization because they can challenge the status quo and genuinely share their ideas without being affected by opposition and disapproving reactions. A recent Chinese study, Zhou et al. [43], found that high motivation to make suggestions and a sense of belonging to the organization account for why voice behavior is typical among nurses with high job embeddedness. This study also provides empirical support for the significant negative relationship between employee voice and intention to emigrate. Based on the knowledge of the authors, there is a lack of previous studies on this relationship. However, nurses want to perceive that their voices are heard, their opinions are valued, and their inputs are embraced [97]. When these perceptions are heightened, nurses may become strongly connected to their organizations, lessening their intention to emigrate.

Furthermore, another important finding of this study is that employee engagement did not mediate the relationship between job embeddedness and intention to emigrate (H3). This was contrary to the expectation of this study. This means the high engagement observed among nurses who are highly connected to their coworkers, community, and employing healthcare organization will not translate into low emigration intention. This addresses the fact that other mediators may be strong enough to reduce the independent contribution of employee engagement when investigated alone as a mediator. To fully realize the mediation role of employee engagement, other potential mediators, for example, voice (as considered in this study), must be in the interplay. Additionally, part of the mediation effect further revealed that job embeddedness has a positive and significant relationship with employee engagement. This finding is in line with the study of Shibiti et al. [98]. Attributes of employees with high embeddedness (deep link and good fit) are resources that allow employees to develop a feeling of purpose and meaning, birthing increasing levels of engagement. Furthermore, another part of the mediation effect established that employee engagement has a negative and significant relationship with intention to emigrate. This result appears to support the current attention in the retention literature [15, 99]. This shows that employee engagement can mitigate nurses' emigration intention by fostering a more enriching work experience that lessens exit.

Lastly, a serial mediator model was developed in this study stating that both employee voice and engagement can serially mediate the negative relationship between job embeddedness and intention to emigrate (H4). The result revealed that the combination of the two mediators made the job embeddedness and intention to emigrate significant. The importance of this result is encapsulated in the complexity of emigration which has a web of connections with pull and push factors and international recruitment [100]. Thus, a call is made for future studies to identify significant factors that influence nurses' emigration intentions. Considering that there is a modest amount of empirical studies about job embeddedness and intention to emigrate relationship, the finding of this study offers a significant contribution to the body of knowledge.

## 5. Conclusion

### 5.1. Contribution to Research

The findings of the study extend research on employee retention by integrating job embeddedness into the traditional emigration intention model. This model offers a subtle perspective on the factors affecting the decision of employees to emigrate, mainly by elucidating the paradoxically overlooked “pull” factors that draw employees to new prospects abroad. While extant studies have paid much attention to “push” factors, this study underscores the importance of knowing the conditions that attract employees to make emigration decisions. In this regard, the study provides a detailed insight into emigration intentions, filling a critical gap and providing an empirical understanding for healthcare organizations aiming to retain their skilled healthcare professionals. The findings of this study established the relationship between job embeddedness and emigration intention in the context of a low-income country with the support of Lewin's field theory and the theory of cumulative causation of migration. This rarely explored relationship reveals that nurses with high job embeddedness can be pulled to remain in their home country, while low job embeddedness can push nurses to emigrate in the quest for better opportunities abroad. On the other hand, the findings of this study further illustrate the role job embeddedness can play in reducing emigration intention sponsored by networks and social structures, as elucidated by the theory of cumulative causation of migration. Highly embedded employees will likely perceive emigration as a factor that would break their links with the organization and community. When they are surrounded by the pull factors, networks and social structures that ease the emigration process, they may not nurture the intention to emigrate as long as they perceive a good fit between their expectation and what their organization and community offer [101]. This study also considered workplace attitudes and behaviors such as employee voice and engagement, which are more impactful in the present complex and dynamic healthcare environment. Accordingly, this study contributes to the brain drain literature. In essence, it can be stated that this study presents new proof of the nexus between the investigated variables and substantiates the existing relationship between them. Additionally, this study adds to the voice and engagement literature. Coupled with the understanding that organization should be concerned with the voice and engagement of their employees, this study apparently supports the need for organizations to emphasize employee voice and engagement as mediators for job embeddedness and intention to emigrate relationship. Hopefully, hospital managers can make a pursuit to foster these two variables to unravel the complexity behind how nurses can be retained and motivated to positively impact healthcare service delivery.

### 5.2. Implication

The logical inconsistencies of the theory of cumulative causation of migration exist despite its strong theoretical foundation. One of the inconsistencies is that its circular nature prevents it from properly identifying contextual feedback mechanisms that would halt or slow the internal self-reinforcing dynamics of emigration [102]. While job embeddedness is considered a retention-related psychological state and not being feedback mechanism in the traditional sense, it has been reported to be shaped by contextual feedback such as quality of work life [103] and recognition [104]. However, without mincing words, the theory of cumulative causation of migration has been extended through this study because this study has elucidated how nurses' job embeddedness creates a micro-level retention mechanism that interrupts migration patterns. The study shows how workplace-based psychological, professional, and positive organizational behavior decreases emigration intention. This study, therefore, offers an empirical extension to the need for refinement of the theory of cumulative causation of migration with personal-level anchors in its cumulative feedback process.

The study extends Lewin's field theory by showing that job embeddedness can act as a dynamic variable whose retention power hinges on its potency for voice behavior among employees. The serial mediation shows that job embeddedness can directly and indirectly reduce workplace retention issues. It also shows that engagement fails to counteract emigration pressures unless voice empowerment occurs first. This suggests that engagement may not often be an independent mediator in retention studies. The study extends this theory by showing that voice expression is a latent mechanism that suppresses migration feedback loops among embedded nurses.

The findings of this study confirm that employees will likely not display intention to emigrate if they have deep links and good fits with their organization. Often, this high embeddedness leads to a high cost of leaving the organization, thereby decreasing the likelihood of nurses' emigration intention. Increasing job embeddedness among nurses may yield a beneficial consequence for both healthcare employers and employees. The healthcare sector is experiencing obvious changes due to emigration challenges, digitalization, and budget constraints; in these impulsive changes, there is an urgent need for healthcare managers to nurture increasing job embeddedness so as to help the system to remain resilient in the shifting landscapes. To foster connections between nurses, community, and their job, HR managers can sponsor programs, policies, outreaches, and interprofessional collaboration. For example, monthly and annual outreach can be organized where nurses are gathered to help people who are poor and have health challenges; interprofessional collaboration can be organized to improve interprofessional interactions, workplaces, health systems, and patient care [105]. Embeddedness initiatives can be overtly focused on to support open communication routes, such as hospital-level nurse advisory councils, ensuring nurses feel heard and valued. Besides, policymakers can establish structural reforms in their job embeddedness strategies by making voice an underlying component instead of using ostensive retention measures. Professional associations like NANNM need to shift their approach from basic advocacy work toward developing specific platforms that support individual nurse voice behavior and transform their embedded connections into substantial engagement. Hence, nurses' perception of workplace security and empowerment will create opportunities to stop the cumulative causation of emigration through addressing its psychosocial origins.

The findings of this study also hold a practical implication aligned with SDGs 3 and 8. By supporting strengthened linkages between employees and their workplaces via relationship-focused leadership, meaningful work, and community engagement, healthcare organizations can retain skilled healthcare professionals, which will contribute to the improvement of the healthcare systems (SDG 3). Also, supporting job embeddedness can lead to having decent work environments and sustainable human resource capacity-building (SDG 8). The study links these findings to SDG 8 (decent work) by establishing that psychological safety is a fundamental requirement for maintaining a sustainable health workforce. This may ascertain that skilled healthcare professionals are readily available to offer quality service delivery, thereby upholding the sustainability of the economy and the well-being of the local people.

### 5.3. Limitations

In this study, a causal inference was not drawn because of the use of cross-sectional approach. Future studies can use a longitudinal design to obtain insight into the evolving nature of job embeddedness and its influence on the transition of employees' emigration intention to action. Besides, this study placed reliance on self-reports of the nurses in respect to the items of the studied variables. Relying on their responses was appropriate because employee behavior is better explained using individual perceptions [106]. Also, this study embraced quantitatively collected data for this study, which could limit the depth of insight regarding the motivations and contributing factors supporting the nurses' emigration decision. Hence, future study should investigate the relationship between job embeddedness and intention to emigrate by using both quantitative and qualitative approach to achieve a good level of robustness. Researchers of this study are aware of the risk associated with using single-source data, which can raise concerns about CMV and exaggerate studied relationships. Future studies can use superiors to report employee voice and engagement. The setting of this study is the Nigerian healthcare system, focusing on nurses. It will be interesting if future studies can sample other healthcare professionals like medical doctors or pharmacists because previous studies have reported high brain drain rates [107, 108]. Additionally, future studies can examine professionals outside the healthcare environment, such as teachers [109], and in other countries with different cultural settings. This will confirm if the findings of this study are generalizable. We used job embeddedness as a whole instead of on-the-job embeddedness and off-the-job embeddedness. Future studies can test the two dimensions of embeddedness on emigration intentions. Lastly, another limitation of this study is the exclusion of more theoretical control variables that may overlap with job embeddedness and could explain significant variance with intention to emigrate. Future studies can control for these variables, such as job satisfaction [110] (Table A1).

## Figures and Tables

**Table 1:** Findings of reliability and convergent validity.

Construct	Items	Factor loading
*Job embeddedness (JE) (α*=0.718*; AVE = 0.639; CR = 0.841)*		
	JE1	0.808
	JE2	0.780
	JE3	0.809

*Employee voice (EV) (α*=0.860*; AVE = 0.642; CR = 0.900)*		
	EV1	0.856
	EV3	0.805
	EV4	0.791
	EV5	0.767
	EV6	0.784

*Employee engagement (EE) (α*=0.831*; AVE = 0.665; CR = 0.888)*		
	EE2	0.758
	EE4	0.855
	EE5	0.838
	EE7	0.806

*Intention to emigrate (IE) (α*=0.707*; AVE = 0.632; CR = 0.837)*		
	IE1	0.731
	IE2	0.814
	IE3	0.835

*Note:α* = Cronbach's alpha.

Abbreviations: AVE, average variance extracted; CR, composite reliability.

**Table 2:** Discriminant validity assessment: findings of Fornell–Larcker criterion.

	**1**	**2**	**3**	**4**

Employee voice [1]	**0.801**			
Intention to emigrate [2]	−0.683	**0.795**		
Job embeddedness [3]	0.541	−0.474	**0.799**	
Employee engagement [4]	0.529	−0.450	0.370	**0.815**

*Note:* The bold values represent the square root of AVE values.

**Table 3:** Discriminant validity assessment: findings of heterotrait–monotrait criterion.

	**1**	**2**	**3**

Employee voice [1]	—		
Intention to emigrate [2]	0.872	—	
Job embeddedness [3]	0.679	0.665	—
Employee engagement [4]	0.623	0.585	0.473

**Table 4:** Path coefficients, significances, and predictive relevance.

Hypothesis	Paths	*β*	*t*	*p* value	Bootstrap 95% CI	
					BCI-LL	BCI-UL
*Control variables' effect*						
	Gender ⟶ intention to emigrate	0.077	0.957	0.169	−0.057	0.210
	Job tenure ⟶ intention to emigrate	−0.051	1.290	0.099	−0.116	0.014
	Education ⟶ intention to emigrate	0.005	0.100	0.460	−0.074	0.083

*Direct and mediation effects*						
H_1_	Job embeddedness ⟶ intention to emigrate	−0.134	2.385	0.009	−0.233	−0.049
	Job embeddedness ⟶ employee voice	0.541	12.357	< 0.001	0.459	0.606
	Employee voice ⟶ intention to emigrate	−0.545	8.849	< 0.001	−0.635	−0.431
H_2_	Job embeddedness ⟶ employee voice ⟶ intention to emigrate	−0.295	7.153	< 0.001	−0.364	−0.230
	Job embeddedness ⟶ employee engagement	0.118	2.259	0.012	0.027	0.202
	Employee engagement ⟶ intention to emigrate	−0.111	2.389	0.008	−0.188	−0.038
	Employee voice ⟶ employee engagement	0.450	7.925	0.000	0.336	0.559
H_3_	Job embeddedness ⟶ employee engagement ⟶ intention to emigrate	−0.013	1.523	0.064	−0.029	0
H_4_	Job embeddedness ⟶ employee voice ⟶ employee engagement ⟶ intention to emigrate	−0.028	2.181	0.015	−0.050	−0.009

*R* ^2^ = 0.293 (employee voice), 0.290 (employee engagement), 0.490 (intention to emigrate)						

*Q* ^2^ = 0.284 (employee voice), 0.129 (employee engagement), 0.217 (intention to emigrate)						

**Table 5:** Descriptive statistics and correlations of variables (*n* = 336).

	**1**	**2**	**3**	**4**	**5**	**6**	**7**	**8**	**9**	**10**	**11**

Gender [1]	1										
Age [2]	0.023	1									
Marital status [3]	−0.014	0.535^∗∗^	1								
Job tenure [4]	−0.073	0.108^∗^	0.118^∗^	1							
Education [5]	−0.116^∗^	−0.214^∗∗^	−0.051	0.334^∗∗^	1						
Hospital type [6]	−0.024	0.026	−0.062	−0.089	−0.045	1					
Service years [7]	0.005	0.090	0.039	0.258^∗∗^	0.068	−0.164^∗∗^	1				
JE [8]	−0.053	−0.020	0.031	0.084	0.161^∗∗^	−0.064	0.027	1			
EV [9]	−0.109^∗^	−0.123^∗^	−0.046	0.075	0.212^∗∗^	−0.063	0.024	0.537^∗∗^	1		
EE [10]	−0.020	−0.161^∗∗^	−0.096	0.025	0.089	0.031	0.039	0.367^∗∗^	0.527^∗∗^	1	
IM [11]	0.110^∗^	0.070	−0.035	−0.112^∗^	−0.164^∗∗^	0.065	−0.068	−0.476^∗∗^	−0.680^∗∗^	−0.449^∗∗^	1
Mean								2.387	2.384	3.673	2.613
Standard deviation								0.717	0.779	0.706	0.993

*Note:* Gender (1: male; 2: female); job tenure (1: Nurse, 2: Nurse practitioner, 3: Chief Nurse, and 4: Associate Chief Nurse); education (1: Diploma in Nursing, 2: Baccalaureate Technical, 3: Technique Superior, 4: License Technique, 5: Bachelor of Sciences, and 6: Master's degree).

^∗^ *p* < 0.05.

^∗∗^ *p* < 0.01.

**Table 6:** Measurement of constructs.

Variable Items
Job embeddedness
I am too caught up in this hospital to leave
I feel tied to this hospital
I am tightly connected to this hospital
Employee voice (which reflects unit-focused or organizationally focused behavior that is positive but more challenging in nature)
I develop and make recommendations concerning issues that affect this work group.
I speak up and encourage others in this group to get involved in issues that affect the group.
I communicate my opinions about work issues to others in this group even if my opinion is different and others in the group disagree with me.
I am well informed about issues where my opinion might be useful to this work group.
I get involved in issues that affect the quality of work life here in this group.
I speak up in this group with ideas for new projects or changes in procedures.
Employee engagement
At my work, I feel bursting with energy.
At my job, I feel strong and vigorous.
When I get up in the morning, I feel like going to work.
I am enthusiastic about my job.
My job inspires me.
I am proud of the work that I do.
I feel happy when I am working intensely.
I am immersed in my work.
I get carried away when I am working.
Intention to change profession
I think a lot about leaving the profession
I am actively looking for another job outside the nursing profession, and
I will leave the nursing profession as soon as possible.
Intention to migrate
I often think about immigrating to another country to live there permanently
I often think about working and living in another country for an extended period of time
I often think about searching for better job prospects abroad

## Data Availability

The data that support the findings of this study are available from the corresponding author upon request.
